# Comparison of Assisted Reproductive Technology (ART) Outcomes in Two Controlled Ovarian Stimulation Protocols Using Follitropin Delta, a Recombinant Follicle-Stimulating Hormone (rFSH) Injection

**DOI:** 10.7759/cureus.81281

**Published:** 2025-03-27

**Authors:** Shoji Kokeguchi, Eri Okamoto, Hiroaki Shibahara, Kazuki Yamagami, Kohyu Furuhashi, Noritoshi Enatsu, Masahide Shiotani

**Affiliations:** 1 Medical Physics, Hanabusa Women's Clinic, Kobe, JPN; 2 Embryology, Hanabusa Women's Clinic, Kobe, JPN

**Keywords:** assisted reproductive technology art, follitropin delta, gnrh antagonist protocol, ppos, recombinant follicle-stimulating hormone

## Abstract

Aim: This study aimed to compare the outcomes of assisted reproductive technology (ART) between progestin-primed ovarian stimulation (PPOS) and antagonist protocols, using follitropin delta as the sole ovarian stimulation agent. While many comparative studies on PPOS and antagonist protocols exist, most utilize follitropin alpha or beta as stimulatory agents. Notably, no studies have reported on the use of follitropin delta in this context.

Methodology: A retrospective analysis was conducted on ART cases initiated in 2022, including 529 PPOS cycles and 298 antagonist cycles. Subgroup analyses were performed based on anti-Müllerian hormone (AMH) levels, dividing patients into four groups: <1.2 ng/mL, 1.2≤ AMH <2.03 ng/mL, 2.03≤ AMH <5.0 ng/mL, and ≥5.0 ng/mL. Assessed outcomes included the number of retrieved oocytes, fertilization rates, cleavage rates, blastocyst formation rates (BL rates), and good-quality blastocyst formation rates (GBL rates), as well as the number of retrieved oocytes, fertilized embryos, cleavage-stage embryos, BL, and GBL. All cases were planned for complete blastocyst vitrification, including those in the antagonist group.

Results: The mean patient age was 35.1 years in the PPOS group and 36.2 years in the antagonist group. Other baseline characteristics included the causes and duration of infertility, proportion of primary infertility, baseline hormone levels, BMI, duration and dosage of follitropin delta administration, and duration of medroxyprogesterone acetate (MPA) use. There were no significant differences in the background characteristics between the two groups. In AMH levels of 2.03 ng/mL and above, the two groups with AMH-based subgroups showed that the PPOS protocol demonstrated a significantly higher number of retrieved oocytes, BL, and GBL compared to the antagonist protocol.

Conclusion: While previous studies have indicated that no significant differences in outcomes between these protocols, the present study observed that the follitropin delta enhanced the formation of blastocysts and good-quality blastocysts. Further research is needed to determine whether these findings are specific to follitropin delta or extend to other recombinant follicle-stimulating hormone (rFSH) preparations.

## Introduction

Initially, in vitro fertilization (IVF) was performed during the natural ovulation cycles [1]. However, IVF procedures have evolved to involve the retrieval of oocytes from multiple well-developed follicles through controlled ovarian stimulation (COS). The advancement of COS highlighted a nearly 20% risk of premature ovulation or early luteinization without the suppression of large developing follicles, often leading to cycle cancellation. To address this issue, gonadotropin-releasing hormone (GnRH) agonists were developed [2]. In the 1980s, protocols using GnRH agonists to suppress premature luteinizing hormone (LH) surges were introduced, significantly reducing cycle cancellation rates and establishing a reliable method for oocyte retrieval [2]. In the late 1990s, GnRH antagonists were introduced to clinical practice. These antagonists have rapidly become indispensable in ovarian stimulation protocols due to their ability to mitigate the transient gonadotropin flare-up effect associated with GnRH agonists. In 2015, Kuang et al. [3] reported on the progestin-primed ovarian stimulation (PPOS) protocol, which leveraged the ability of progestins to suppress LH surges. The PPOS protocol offers several advantages over the antagonist method, including lower cost, reduced injection burden, and a potentially lower risk of ovarian hyperstimulation syndrome (OHSS). However, the PPOS protocol also has limitations such as the inability to perform fresh embryo transfers and the necessity for complete embryo vitrification, which may extend the time to pregnancy. In Japan, complete embryo vitrification without fresh embryo transfer has become standard practice, aligning with the PPOS protocol. In Japan, recombinant follicle-stimulating hormone (rFSH) is the primary injectable medication used in IVF. Recombinant FSH plays a central role in reproductive medicine and has evolved with changing approaches to control ovarian stimulation. Early protocols emphasized maximum stimulation to achieve the highest possible oocyte yield; however, the focus has shifted toward individualized stimulation to achieve an optimal yield. Although no single algorithm can account for all factors that influence dosage determination, anti-Müllerian hormone (AMH) levels are considered critical [4].

Follitropin delta differs from traditional rFSH preparations as it is derived from human cells and features a unique dosing algorithm based on AMH levels and body weight [5,6]. Studies by Ishihara et al. demonstrated that compared to follitropin beta, follitropin delta is associated with a lower incidence of OHSS [7,8]. Considering these characteristics, follitropin delta was selected for this study. This investigation focused exclusively on assisted reproductive technology (ART) outcomes stratified by AMH levels, representing ovarian reserve, using follitropin delta as the sole injectable agent administered according to AMH- and weight-based dosing algorithms.

## Materials and methods

Study population

This study was conducted at a facility in Japan treating patients undergoing ART under the public health insurance system implemented in 2022. A total of 529 cycles using the PPOS protocol and 298 cycles using the antagonist protocol were analyzed. The inclusion criteria were as follows: (1) women diagnosed as infertile without additional complications, (2) women aged between 28 and 42 years at the time of oocyte retrieval, (3) patients who had undergone ART with up to two oocytes picked up before, and (4) oocyte retrieval performed by a skilled physician at our facility.

Patients were stratified into four subgroups, Group A to Group D, based on AMH levels as follows: (1) Group A: Patients with diminished ovarian reserve (AMH <1.2 ng/mL), (2) Group B: Patients with intermediate-low AMH (1.2≤ AMH <2.03 ng/mL), (3) Group C: Patients with intermediate-high AMH (2.03≤ AMH <5.0 ng/mL), and (4) Group D: Patients with high ovarian reserve, potentially PCOS (AMH ≥5.0 ng/mL).

A cutoff value of 2.03 ng/mL, corresponding to 15 pmol/mL in previous studies, was selected for stratification [9]. The <1.2 ng/mL group (Group A) included all cases with AMH levels ≥0.5 ng/mL. The assessed outcomes included the number of retrieved oocytes, fertilization rate, cleavage rate, blastocyst formation rate, and good-quality blastocyst formation rate (GBL rate). The primary endpoint was the ongoing pregnancy rate. For all cycles, including those using the antagonist protocol, the stimulation protocol involved complete blastocyst vitrification, with no fresh embryo transfers. The exclusion criteria were as follows: (1) patients with AMH levels <0.5 ng/mL, (2) patients diagnosed with primary ovarian insufficiency (POI), and (3) cases using a mild stimulation protocol.

COS protocols

In the PPOS group, there were 126 cases in the AMH <1.2 ng/mL group, 138 cases in the 1.2≤ AMH <2.03 ng/mL group, 158 cases in the 2.03≤ AMH <5.0 ng/mL group, and 107 cases in the ≥5.0 ng/mL group. In the antagonist group, there were 63 cases in the AMH <1.2 ng/mL group, 63 cases in the 1.2≤ AMH <2.03 ng/mL group, 144 cases in the 2.03≤ AMH <5.0 ng/mL group, and 28 cases in the AMH ≥5.0 ng/mL group.

In the PPOS protocol, medroxyprogesterone acetate (MPA) (5 mg/day) was administered orally from day three of the menstrual cycle until the day before oocyte retrieval. COS was performed using follitropin delta (Rekovelle, Ferring Pharmaceuticals) with dosing based on a fixed algorithm without adjustments during the stimulation cycle. A GnRH agonist flare protocol was used for oocyte retrieval. Buserelin nasal spray (0.15%, 10 mL) was administered at 300 µg per spray in each nostril at 19:00 and 20:00 on the day of the trigger. Alternatively, dual triggering with 5,000 IU of human chorionic gonadotropin (hCG) (hCG5000(F), Fuji Pharma) or Ovitrelle Pen (250 µg, Merck Serono) was used if the flare was ineffective in a previous cycle. Follicular development was monitored by transvaginal ultrasound. When dominant and secondary follicles exceeded 18 mm in diameter, estradiol, LH, and progesterone levels were measured multiple times to confirm readiness for oocyte retrieval. Oocyte retrieval was performed between 8:30 AM and 9:30 AM on the day after triggering.

In the antagonist protocol, follitropin delta was used for ovarian stimulation. GnRH antagonist suppression was achieved with ganirelix acetate (Ganirest, Organon) at a flexible dose of 0.25 mg/day by subcutaneous injection. The method for determining the oocyte retrieval timing was identical to that of the PPOS protocol.

After oocyte retrieval, insemination was performed by intracytoplasmic sperm injection (ICSI), conventional IVF, or the split method. Blastocyst development was evaluated five and six days after retrieval. The quality of blastocysts was graded using the Gardner classification, with good-quality blastocysts defined as those graded ≥3 without a C rating for inner cell mass (ICM) or trophectoderm (TM). Embryo transfer after oocyte retrieval was performed using frozen-thawed embryo transfer (FET) after a waiting period of one cycle. This procedure was conducted following a hormone replacement therapy (HRT) protocol. Clinical pregnancy was defined as the confirmation of a gestational sac via transvaginal ultrasonography. Furthermore, clinical pregnancy was defined as the presence of a fetal heartbeat confirmed at around six to seven weeks of gestation, with the pregnancy continuing beyond 12 weeks.

Statistical analysis

For the background, since the data is non-parametric, the Mann-Whitney U test was used, and values were expressed as medians (minimum-maximum). Statistical significance was set at P<0.05. For the specific parameters of clinical pregnancy rate, ongoing pregnancy rate, abnormal fertilization, and abnormal cleavage, Pearson's chi-square test was performed. For non-normally distributed quantitative variables, the Mann-Whitney U test was used; for normally distributed variables with equal variance, Student’s t-test was applied, and Welch’s t-test was used for those with unequal variance. To determine whether the PPOS or antagonist protocol contributes more to the dependent variable, additional tests were conducted in groups B-D with AMH ≥1.2 ng/mL, where significant differences were observed in the previous analysis. The number of oocytes retrieved, the number of blastocysts formed, and the number of good-quality blastocysts were analyzed using multiple regression analysis. The significance level was set at P<0.05. Odds ratios and 95% CIs were calculated using multivariate logistic regression analysis, adjusting for PPOS or antagonist protocol, age, AMH levels, weight, and BMI.

AMH measurement

AMH levels were measured using an electrochemiluminescence immunoassay (ECLIA) on a Cobas e411 immunoassay analyzer with Elecsys AMH Plus reagent.

## Results

Background

Patient characteristics are summarized in Table 1. The mean ages were 35.1 years in the PPOS group and 36.2 years in the antagonist group. The median age of the PPOS group was 39 years, while that of the antagonist group was 38 years; however, there was no significant difference between the two groups. There were no significant differences between the groups in terms of causes of infertility, duration of infertility, proportion of primary infertility, baseline hormone levels, BMI, duration and dosage of follitropin delta administration, or duration of MPA use. The median weight was 53.0 kg for the PPOS group and the same for the antagonist group. The median BMI was 20.9 for both groups, showing no significant differences between them. The two groups had similar baseline characteristics, including infertility duration, history of pregnancy, and history of abortion.

**Table 1:** Background and reproductive characteristics of women undergoing ovarian stimulation with follitropin delta All variables were non-normally distributed and analyzed using the Mann-Whitney U test. Stimulation with the PPOS protocol and the antagonist protocol group showed no significant differences in serum AMH level, body weight, BMI, duration of infertility, number of pregnancies, and number of abortions. AMH, anti-Müllerian hormone; PPOS, progestin-primed ovarian stimulation

		PPOS				Antagonist				
	(n)	Minimum	Median	Maximum	(n)	Minimum	Median	Maximum	z-value	P-value
Age all	529	28	39	42	298	28	38	42	1.42	0.16
Category										
<35	223	28	32	34	93	28	32	34	0.50	0.61
35-37	115	35	36	37	45	35	36	37	1.44	0.15
38-40	127	38	39	40	98	38	39	40	-0.61	0.54
41-42	64	41	42	42	62	41	42	42	0.07	0.93
AMH-based groups										
<1.2	138	28	35	42	63	28	38	42	-1.29	0.19
1.2<2.03	158	28	34	42	144	28	37	42	-1.93	0.06
2.03<5.0	107	28	32	41	28	28	33	40	-1.75	0.07
5.0≤	223	0.5	2.4	22.0	298	0.5	2.2	14.9	1.77	0.07
AMH (ng/mL) all										
<1.2	126	0.5	0.82	1.19	126	0.5	0.74	1.19	-1.44	0.15
1.2<2.03	138	1.2	1.69	2.01	138	1.2	1.68	2.01	-1.80	0.07
2.03<5.0	158	2.03	3.18	4.96	158	2.03	2.36	4.96	-1.61	0.10
5.0≤	107	5.01	6.43	22.15	107	5.04	6.18	14.5	0.42	0.67
Weight (kg)	529	35.7	53.0	88.0	298	32.0	53.0	86.6	-1.46	0.14
BMI	529	15.4	20.9	36.8	298	15.0	20.9	33.9	-0.52	0.60
Duration of infertility (m)	529	6	12	60	298	6	17	60	-1.24	0.12
No of pregnancy	529	0	0	2	298	0	0	4	-1.34	0.09
No of abortion	529	0	0	3	298	0	0	4	-1.26	0.13

COS, oocyte retrieval, and subsequent culture outcomes stratified by AMH subgroups are summarized for the PPOS protocol (Table 2) and the antagonist protocol (Table 3). Across both groups, the duration of rFSH administration, MPA use, and daily FSH dosage were comparable. Indicators assessed at the time of oocyte retrieval included the number of retrieved oocytes, degenerated oocytes, retrieval rates per punctured follicle, and the number of metaphase II (MII) oocytes in cases using ICSI. The distribution of ART methods, including IVF, ICSI, or the split method, was similar across the groups. The outcome metrics included the number of fertilized oocytes, cleaved embryos, blastocyst rates, and good-quality blastocyst rates.

**Table 2:** Duration of ovarian stimulation and total dosage of follitropin delta administered with the PPOS protocol, along with culture outcomes stratified by AMH subgroups A: The culture results of the number of retrieved oocytes, fertilized eggs, cleavaged eggs, blastocyst, and good blastocyst. B: The culture results of average±SD (%) of cases were calculated. C: The culture results, represented as the number of cases divided by the total number of cases. Group A: Patients with diminished ovarian reserve (AMH <1.2 ng/mL). Group B: Patients with intermediate-low AMH (1.2≤ AMH <2.03 ng/mL). Group C: Patients with intermediate-high AMH (2.03≤ AMH <5.0 ng/mL). Group D: Patients with high ovarian reserve or potential PCOS (AMH ≥5.0 ng/mL). SD, standard deviation; AMH, anti-Müllerian hormone; PPOS, progestin-primed ovarian stimulation; rFSH, recombinant follicle-stimulating hormone; MPA, medroxyprogesterone acetate; ART, assisted reproductive technology; ICSI, intracytoplasmic sperm injection; IVF, in vitro fertilization

	PPOS (n=529）				
	AMH subgroup	Group A	Group B	Group C	Group D
	Number	126	138	158	107
	rFSH administration( days±SD)	8.0±1.6	8.2±1.3	8.5±1.2	8.7±1.2
	MPA administration (days±SD)	7.4±1.6	7.6±1.2	7.7±1.3	8.2±1.3
	Thickness of endometrium (mm±SD)	7.2±1.7	7.9±2.0	7.3±1.8	8.1±1.7
	rFSH dosage (μg/day±SD)	11.8±0.2	11.9±0.4	10.7±2.2	7.2±1.9
	ART method				
	ICSI (n)	74	78	146	55
	IVF (n)	25	15	16	5
	Split (n)	27	45	79	47
A	No. of retrieved oocytes (n±SD）	4.3±3.4	7.5±4.0	9.5±5.4	12.7±7.0
	No. of fertilized eggs (n±SD）	3.1±2.3	4.9±3.1	6.1±4.1	7.9±4.6
	No. of cleavaged eggs (n±SD）	3.0±2.2	4.4±2.9	5.7±3.8	7.3±4.4
	No. of blastocysts (n±SD）	2.1±1.6	2.9±2.3	3.6±2.7	5.0±3.5
	No. of good blastocyst (n±SD）	1.5±1.4	1.8±1.5	2.4±2.0	3.3±2.6
B	Fertilized rate (%±SD）	70.4±29.8	66.2±25.0	63.2±22.9	62.1±22.6
	Cleavaged rate (%±SD）	91.7±19.5	85.9±24.2	93.3±11.7	92.0±14.5
	Blastocyst rate (%±SD）	60.1±35.9	53.0±32.0	58.5±29.9	63.0±22.3
	Good blastocyst rate (%±SD）	42.9±36.6	34.0±26.8	39.9±29.0	41.0±25.3
C	Fertilized rate (%）	67.8	65	63.5	61.3
	Cleavaged rate (%）	92.9	89	92.8	92.9
	Blastocyst rate (%）	60.1	56.6	59.2	63.8
	Good blastocyst rate (%）	42.0	35.8	39.6	42.1

**Table 3:** Duration of ovarian stimulation, total dosage of follitropin delta administered with the antagonist protocol, and culture outcomes stratified by AMH subgroups A: The culture results of the number of retrieved oocytes, fertilized eggs, cleavaged eggs, blastocyst, and good blastocyst. B: The culture results of average±SD (%) of cases were calculated. C: The culture results, represented as the number of cases divided by the total number of cases. Group A: Patients with diminished ovarian reserve (AMH <1.2 ng/mL). Group B: Patients with intermediate-low AMH (1.2≤ AMH <2.03 ng/mL). Group C: Patients with intermediate-high AMH (2.03≤ AMH <5.0 ng/mL). Group D: Patients with high ovarian reserve or potential PCOS (AMH ≥5.0 ng/mL). SD, standard deviation; AMH, anti-Müllerian hormone; PPOS, progestin-primed ovarian stimulation; rFSH, recombinant follicle-stimulating hormone; MPA, medroxyprogesterone acetate; ART, assisted reproductive technology; ICSI, intracytoplasmic sperm injection; IVF, in vitro fertilization

Antagonist (n=298)					
	AMH subgroup	Group A	Group B	Group C	Group D
	Number	63	63	144	28
	rFSH administration (days±SD)	7.2±1.9	7.3±1.4	8.6±2.0	8.8±0.8
	MPA administration (days±SD)				
	Thickness of endometrium (mm±SD)	9.1±1.9	9.3±1.9	9.3±2.1	9.9±1.8
	rFSH dosage (μg/day±SD)	12.0±0.0	11.5±1.0	10.8±1.8	6.6±1.7
	ART method	45	42	88	16
	ICSI (n)	12	6	22	4
	IVF (n)	6	15	34	8
	Split (n)	1	1	2	0
A	No. of retrieved oocytes (n±SD）	3.9±3.3	6.1±4.0	7.7±4.3	9.7±5.2
	No. of fertilized eggs (n±SD）	2.6±2.3	4.1±2.8	4.9±3.3	6.3±3.1
	No. of cleavaged eggs (n±SD）	2.3±2.2	3.6±2.6	4.4±3.1	5.7±2.6
	No. of blastocysts (n±SD）	1.3±1.7	2.0±2.1	2.3±2.4	2.7±2.5
	No. of good blastocyst (n±SD）	0.8±0.9	1.2±1.3	1.4±1.6	1.3±1.1
B	Fertilized rate (%±SD）	67.1±32.6	69.4±29.6	65.7±25.3	70.4±21.9
	Cleavaged rate (%±SD）	91.9±19.4	89.6±18.9	87.8±20.3	91.7±13.7
	Blastocyst rate (%±SD）	47.8±41.1	41.1±29.9	40.8±32.6	40.7±34.3
	Good blastocyst rate (%±SD）	33.9±38.7	25.7±26.0	26.2±26.8	22.6±22.7
C	Fertilized rate(%）	66.9	66.8	64.3	65.3
	Cleavaged rate (%）	90.9	88.4	88.5	89.9
	Blastocyst rate (%）	50.6	49.2	46.7	43.5
	Good blastocyst rate (%）	30.1	29.2	28.6	21.2

Oocyte retrieval count

In the <1.2 ng/mL AMH group, the average number of retrieved oocytes for the PPOS protocol was 4.3, and for the antagonist protocol, it was 3.9, with no significant difference. In the 1.2-2.03 ng/mL AMH group, the numbers of retrieved oocytes were 7.5 and 6.1, respectively, while in the 2.03-5.0 ng/mL group, they were 9.5 and 7.7, showing a significant difference in favor of the PPOS protocol. In the >5.0 ng/mL group, the values were 12.7 and 9.7, respectively, again favoring the PPOS protocol (Table 4).

**Table 4:** Comparison of the number of retrieved oocytes, blastocyst formations, and good-quality blastocysts between the PPOS protocol and the antagonist protocol For non-normally distributed quantitative variables, the Mann-Whitney U test was used. For normally distributed variables with equal variance, Student’s t-test was applied, and for those with unequal variance, Welch’s t-test was used. Group A: Patients with diminished ovarian reserve (AMH <1.2 ng/mL). Group B: Patients with intermediate-low AMH (1.2≤ AMH <2.03 ng/mL). Group C: Patients with intermediate-high AMH (2.03≤ AMH <5.0 ng/mL). Group D: Patients with high ovarian reserve or potential PCOS (AMH ≥5.0 ng/mL). AMH, anti-Müllerian hormone; PPOS, progestin-primed ovarian stimulation

	PPOS vs. antagonist		
AMH <1.2 (Group A)	PPOS	Antagonist	P-value
No. of retrieved oocyte (n±SD）	4.3±3.4	3.9±3.3	0.40
No. of blastocyst (n±SD）	2.1±1.6	1.3±1.7	0.806
No. of good blastocyst (n±SD）	1.5±1.4	0.8±0.9	0.08
AMH 1.2~2.03 (Group B)	PPOS	Antagonist	P-value
No. of retrieved oocyte (n±SD）	7.5±4.0	6.1±4.0	0.03
No. of blastocyst (n±SD）	2.9±2.3	2.0±2.1	0.23
No. of good blastocyst (n±SD）	1.8±1.5	1.2±1.3	0.13
AMH 2.03~5.0 (Group C)	PPOS	Antagonist	P-value
No. of retrieved oocyte (n±SD）	9.5±5.4	7.7±4.3	<0.0001
No. of blastocyst (n±SD）	3.6±2.7	2.3±2.4	<0.0001
No. of good blastocyst (n±SD）	2.4±2.0	1.4±1.6	<0.0001
AMH ≥5.0 (Group D)	PPOS	Antagonist	P-value
No. of retrieved oocyte (n±SD）	12.7±7.0	9.7±5.2	0.02
No. of blastocyst (n±SD）	5.0±3.5	2.7±2.5	0.002
No. of good blastocyst (n±SD）	3.3±2.6	1.3±1.1	<0.0001

Blastocyst formation count

For the <1.2 ng/mL AMH group, the average number of blastocysts was 2.1 for the PPOS protocol and 1.3 for the antagonist protocol, with no significant difference. In the 1.2-2.03 ng/mL group, the numbers were 2.9 and 2.0, respectively, with no significant difference. In the 2.03-5.0 ng/mL group, they were 3.6 and 2.3, showing a significant difference in favor of the PPOS protocol. In the >5.0 ng/mL group, the numbers were 5.0 and 2.7, respectively, indicating significantly more blastocysts with the PPOS protocol (Table 4).

Good blastocyst count

In the <1.2 ng/mL AMH group, the average number of good-quality blastocysts was 1.5 for the PPOS protocol and 0.8 for the antagonist protocol, showing no significant difference. In the 1.2-2.03 ng/mL group, the numbers were 1.8 and 1.2, respectively, while in the 2.03-5.0 ng/mL group, they were 2.4 and 1.4, showing a significant difference in favor of the PPOS protocol. In the >5.0 ng/mL group, the numbers were 3.3 and 1.3, respectively, again favoring the PPOS protocol (Table 4).

Pregnancy rates and ongoing pregnancy rates

There were no significant differences in pregnancy rates or ongoing pregnancy rates between the PPOS and antagonist protocols (Table 5). The pregnancy rates in each AMH subgroup gradually increased with increasing AMH levels. In the AMH ≥ 5.0 ng/mL group, the pregnancy rate reached approximately 30% (Table 5).

**Table 5:** Comparison of clinical pregnancy rate and ongoing pregnancy rate between PPOS protocol and antagonist protocol. Pearson's chi-square test was performed for statistical analysis.

		PPOS	Antagonist	P-value	odds
Clinical pregnancy rate					
AMH	~1.2(Group A)(%)	24.4	18.1	0.31	1.45
AMH	1.2~2.03(GroupB)(%)	28.3	27.1	0.17	1.0
AMH	2.03~5.0(GroupC)(%)	33.3	31.1	0.65	1.1
AMH	5.0~(GroupD)(%)	41.8	35.8	0.5	1.28
Ongoing pregnancy rate					
AMH	~1.2(GroupA)(%)	20.9	13.6	0.2	1.68
AMH	1.2~2.03(GroupB)(%)	25.1	23.4	0.77	1.09
AMH	2.03~5.0(GroupC)(%)	29.5	23.3	0.18	1.37
AMH	5.0~(GroupD)(%)	37.5	28.2	0.27	1.53

Abnormal fertilization and abnormal developmental growth were defined as cases where fertilization did not occur despite IVF or ICSI, and where cleavage arrest or failure to develop into a blastocyst was observed.

In the <1.2 ng/mL AMH group, the rate of abnormal growth was 12.5% for the PPOS protocol and 25.7% for the antagonist protocol, with a significant difference (P=0.01). In the 1.2-2.03 ng/mL group, the rates were 19.3% and 11.1%, respectively. In the 2.03-5.0 ng/mL group, the rates were 10.3% and 13.3%, respectively, with no significant difference observed. In the >5.0 ng/mL group, the rates were 7.0% for the PPOS protocol and 10.2% for the antagonist protocol, with no significant difference noted (Table 6).

**Table 6:** Comparison of abnormal fertilization and abnormal growth between the PPOS protocol and the antagonist protocol, stratified by AMH subgroups Pearson's chi-square test was used for statistical analysis. AMH, anti-Müllerian hormone; PPOS, progestin-primed ovarian stimulation

	Abnormal fertilization		PPOS, % (n)	Antagonist, % (n)	P-value	Odds
	AMH	~1.2	2.0% (3/143)	1.5% (1/66)	0.77	0.71
	AMH	1.2~2.03	1.2% (2/155)	1.2% (1/81)	0.97	0.95
	AMH	2.03~5.0	1.6% (3/183)	1.1% (2/180)	0.66	0.67
	AMH	5.0~	1.4% (2/141)	0% (0/39)	0.45	0
	Abnormal growth		PPOS	Antagonist	P-value	Odds
	AMH	~1.2	10.4% (15/143)	24.2% (16/66)	0.01	2.73
	AMH	1.2~2.03	18.0% (28/155)	9.8% (8/81)	0.09	0.49
	AMH	2.03~5.0	8.7% (16/183)	12.2% (22/180)	0.27	1.45
	AMH	5.0~	5.6% (8/141)	10.2% (4/39)	0.31	1.9

Logistic regression analysis evaluating the impact of PPOS protocol using follitropin delta

To determine whether the PPOS or the antagonist protocol contributes more to the dependent variable, additional tests were conducted in groups B-D with AMH ≥1.2 ng/mL, where significant differences were observed in the previous analysis. The number of oocytes retrieved, the number of blastocysts formed, and the number of good-quality blastocysts were analyzed using multiple regression analysis. The significance level was set at P<0.05. Odds ratios and 95% CI were calculated using multivariate logistic regression analysis, adjusting for the PPOS or antagonist protocol, age, AMH levels, weight, and BMI. The PPOS method and AMH level were found to be statistically significant explanatory variables in the above three groups (Table 7).

**Table 7:** Logistic regression analysis evaluating the impact of the PPOS protocol versus the antagonist protocol using follitropin delta β represents the standardized regression coefficient. To assess whether the PPOS or antagonist protocol contributes more to the dependent variable, additional tests were performed in groups B-D with AMH ≥1.2 ng/mL, where significant differences were observed in the previous analysis. The number of oocytes retrieved, the number of blastocysts formed, and the number of good-quality blastocysts were analyzed using multiple regression analysis. The significance level was set at p<0.05. Odds ratios and 95% CIs were calculated using multivariate logistic regression analysis, adjusting for the PPOS or antagonist protocol, age, AMH levels, weight, and BMI. The PPOS method was found to be a statistically significant explanatory variable in all three groups. AMH: anti-Müllerian hormone, SE: standard error

AMH 1.2-2.03, no. of oocyte					
	β	SE	P-value	Lower 95% CI	Upper 95% CI
Age	-0.0785	0.0727	0.2819	-0.2219	0.0649
Weight	0.1163	0.0763	0.1290	-0.0342	0.2667
BMI	-0.4467	0.2007	0.0272	-0.8426	-0.0509
AMH ng/mL	3.2379	1.2569	0.0107	0.7591	5.7168
PPOS	1.3377	0.6003	0.0270	0.1538	2.5216
AMH 2.03-5.0, no. of oocyte					
	β	SE	P-value	Lower 95% CI	Upper 95% CI
Age	-0.1959	0.0597	0.0011	-0.3132	-0.0785
Weight	0.1443	0.0649	0.0268	0.0167	0.2720
BMI	-0.4313	0.1727	0.0130	-0.7710	-0.0916
AMH ng/mL	0.8798	0.3153	0.0055	0.2597	1.4998
PPOS	1.9793	0.5199	0.0002	0.9570	3.0016
AMH 2.03-5.0, no. of blastocyst					
	β	SE	P-value	Lower 95% CI	Upper 95% CI
Age	-0.0906	0.0331	0.0065	-0.1557	-0.0255
Weight	0.0430	0.0360	0.2330	-0.0278	0.1139
BMI	-0.0775	0.0959	0.4193	-0.2660	0.1110
AMH ng/mL	0.4713	0.1750	0.0074	0.1273	0.8154
PPOS	1.2907	0.2885	1.02E-05	0.7235	1.8580
AMH 2.03-5.0, no. of GBL					
	β	SE	P-value	Lower 95% CI	Upper 95% CI
Age	-0.0452	0.0241	0.0610	-0.0925	0.0021
Weight	0.0290	0.0262	0.2690	-0.0225	0.0804
BMI	-0.0503	0.0696	0.4701	-0.1872	0.0866
AMH ng/mL	0.3000	0.1271	0.0188	0.0501	0.5499
PPOS	1.0224	0.2096	1.57E-06	0.6103	1.4344
AMH 5.0-, no. of oocyte					
	β	SE	P-value	Lower 95% CI	Upper 95% CI
Age	-0.0800	0.1465	0.5862	-0.3699	0.2100
Weight	-0.2342	0.1424	0.1025	-0.5161	0.0476
BMI	0.2589	0.3750	0.4912	-0.4831	1.0009
AMH ng/mL	0.9647	0.1837	6.04E-07	0.6012	1.3281
PPOS	2.9048	1.2877	0.0258	0.3569	5.4526
AMH 5.0-, no of blastocyst					
	β	SE	P-value	Lower 95% CI	Upper 95% CI
Age	0.0755	0.0802	0.3478	-0.0831	0.2341
Weight	-0.0631	0.0779	0.4192	-0.2173	0.0910
BMI	0.2003	0.2051	0.3306	-0.2055	0.6062
AMH ng/mL	0.2977	0.1005	0.0036	0.0989	0.4965
PPOS	2.2148	0.7043	0.0021	0.8213	3.6084
AMH 5.0-, no. of GBL					
	β	SE	P-value	Lower 95% CI	Upper 95% CI
Age	0.0103	0.0583	0.8595	-0.1051	0.1258
Weight	-0.0103	0.0567	0.8557	-0.1225	0.1018
BMI	0.0341	0.1493	0.8196	-0.2612	0.3295
AMH ng/mL	0.1536	0.0731	0.0376	0.0090	0.2983
PPOS	1.9756	0.5126	0.0002	0.9615	2.9897

## Discussion

The first baby born via IVF-embryo transfer in 1978 benefited from oocyte retrieval during a natural cycle without ovarian stimulation [1]. Subsequent advancements led to the development of ovarian stimulation protocols. In 1981, clomiphene citrate was introduced, followed by human menopausal gonadotropins, enabling the retrieval of multiple oocytes. The GnRH agonist protocol was established in 1988, and by the 1990s, GnRH antagonist protocols gained traction for effectively preventing OHSS. GnRH antagonists became clinically available in Japan in 2006.

In 2015, the potential for ovarian stimulation during the luteal phase was demonstrated, leading to the adoption of protocols that utilized progestins for complete embryo vitrification cycles. Kuang et al. [4] initially described this approach. Although PPOS protocols vary in the type and dosage of progestins, this study focused on the use of 5 mg/day of MPA. Dong et al. reported that 4 mg/day MPA suppressed ovulation equivalent to 10 mg/day [10].

Additionally, gonadotropin preparations such as HMG and rFSH have been developed for ovarian stimulation. Although definitive guidelines for their specific use remain unclear, studies have demonstrated no significant differences in culture outcomes between rFSH and HMG preparations [11]. Among rFSH variants, follitropin alpha, beta, and delta, follitropin delta stands out for its dosing algorithm based on AMH levels and body weight, enabling individualized stimulation protocols [7].

The latest rFSH preparation, Rekovelle (Ferring Pharmaceuticals), was launched in 2021 and clinical outcomes in Japanese women were published by Ishihara et al. [7]. This study reported that the OHSS incidence and safety outcomes were comparable between follitropin delta and follitropin beta. The differences among follitropin preparations can be attributed to variations in the glycosylation of FSH molecules. Meta-analyses comparing ART outcomes with follitropin delta and alfa have shown that follitropin delta reduces the risk of OHSS in hyper-responders but has comparable outcomes for oocyte retrieval, fertilization rates, blastocyst formation rates, and live birth rates [12]. Haakman et al. analyzed 403 cycles and reported similar rates of good-quality early embryos between follitropin delta and alfa [13]. Currently, there is no definitive conclusion regarding the superiority of one preparation over another.

Regarding ovarian stimulation protocols, studies comparing antagonist and long protocols have found no significant differences in pregnancy rates. However, one study reported a 7% higher ongoing pregnancy and live birth rate with the long protocol [14]. Other studies compared the PPOS and antagonist protocols [15]. In most cases, these two methods yielded comparable outcomes in euploid embryos.

Conversely, some studies suggest potential disadvantages of the PPOS protocol in women aged >38 years, including lower blastocyst formation and euploid embryo rates compared to the antagonist protocol (45.8% vs. 59.9% and 5.4% vs. 26.7%, respectively) [16].

Furthermore, Chen et al. reported that while pregnancy rates were comparable between the PPOS and the antagonist protocols, the antagonist protocol was associated with a shorter time to pregnancy [17]. The study showed the pregnancy rate after a single oocyte retrieval cycle was higher in the antagonist group (36.0%) than in the PPOS group (32.2%), with a shorter mean time to delivery (9.3 months vs. 12.4 months) [17].

Another study [18] reported that oocyte retrieval rates and ongoing pregnancy rates were equivalent between the PPOS and antagonist protocols. Although the PPOS protocol yielded a higher number of retrieved oocytes and MII oocytes, the ongoing pregnancy and live birth rates were similar between the two protocols.

In this study, we compared the overall outcomes of the two protocols and analyzed the results stratified by the AMH subgroup. For the AMH <1.2 ng/mL group, there were no significant differences in outcomes between the PPOS and antagonist protocols. However, in AMH subgroups ≥1.2 ng/mL, the PPOS protocol using follitropin delta resulted in significantly higher oocyte retrieval numbers, blastocyst formation rates, and good blastocyst rates.

A previous study investigating ART outcomes using follitropin alfa and delta, stratified by AMH levels, employed clomiphene/letrozole combined with HMG preparations for ovarian stimulation [19]. This study reported higher clinical pregnancy rates per cycle and cumulative pregnancy rates with follitropin delta compared to follitropin alfa. Additionally, cycle cancellation and early LH surge rates were lower, particularly in low-responder cases using follitropin delta. However, our findings did not reveal significant differences in ART outcomes among low-responder patients using follitropin delta.

Cheus et al. compared the outcomes of the PPOS and antagonist protocols in high-responder cases [20]. Clinical pregnancy rates were 37.5% (147/392) in the PPOS group and 32.7% (128/392) in the antagonist group, with no significant differences observed in the first FET cycle. Additionally, basic research has identified variations in the gene expression of miR-4261 and miR-6989-5p microRNAs related to cell proliferation and apoptosis in the follicular fluid between the PPOS and antagonist protocols. These findings suggest that ovarian stimulation protocols may induce qualitative changes in oocytes [21].

In this study, a higher prevalence of cases failing to reach the blastocyst stage or experiencing developmental arrest during fertilization or cleavage was observed in the antagonist group among low-AMH cases. Regardless of AMH level, the PPOS protocol appears to be an effective treatment option, particularly for cases requiring complete embryo vitrification.

This study was conducted under the premise that ovarian stimulation would be planned for complete embryo freezing using follitropin delta. The objective was to determine how to choose between the antagonist protocol and the PPOS protocol, both of which are currently mainstream methods for COS when using follitropin delta.

When considering the results based on AMH levels, although we did not compare them with previous treatment outcomes in this study, it is speculated that PPOS is less likely to cause fertilization failure or developmental arrest in cases with low AMH levels. Additionally, in cases with normal AMH levels, the PPOS protocol resulted in a higher number of retrieved oocytes and a higher rate of good-quality blastocyst formation. However, pregnancy rates were comparable between the two protocols.

Given these findings, obtaining a higher number of blastocysts may be advantageous for patients who wish to have another child, assuming conception occurs this time, which would involve a series of treatments, including oocyte retrieval, culture, and subsequent FET. If so, the PPOS protocol could offer better prognostic outcomes. However, further studies are needed to confirm this hypothesis.

## Conclusions

The present study observed that follitropin delta enhanced the formation of good-quality blastocysts using the PPOS protocol according to AMH levels. There were no significant differences in clinical and ongoing pregnancy rates between the two protocols. Further research is needed to determine whether these findings are specific to the follitropin delta or extend to other rFSH preparations.
