# Correction: Comparison of Assisted Reproductive Technology (ART) Outcomes in Two Controlled Ovarian Stimulation Protocols Using Follitropin Delta, a Recombinant Follicle-Stimulating Hormone (rFSH) Injection

**DOI:** 10.7759/cureus.c224

**Published:** 2025-05-21

**Authors:** Shoji Kokeguchi, Eri Okamoto, Hiroaki Shibahara, Kazuki Yamagami, Kohyu Furuhashi, Noritoshi Enatsu, Masahide Shiotani

**Affiliations:** 1 Medical Physics, Hanabusa Women's Clinic, Kobe, JPN; 2 Embryology, Hanabusa Women's Clinic, Kobe, JPN

This article has been corrected to include table 5 which was inadvertently omitted from the original version. It has now been included and the tables previously labeled as tables 5 and 6 are now labeled as tables 6 and 7, respectively.

